# Reliability of drilling torque values measured with a novel measurement drill for guiding implant placement protocols

**DOI:** 10.4317/jced.63175

**Published:** 2025-10-01

**Authors:** Yusuke Morimoto, Kazuya Doi, Reiko Kobatake, Kaien Wakamatsu, Tomoko Izumikawa, Yoshifumi Oki, Kazuhiro Tsuga

**Affiliations:** 1 Department of Advanced Prosthodontics, Hiroshima University Graduate School of Biomedical and Health Sciences, Hiroshima, Japan

## Abstract

**Background:**

A novel measurement drill was fabricated to analyze bone density. We hypothesized that evaluating the correlation between primary stability and bone density—assessed using drilling torque values obtained with a novel drill—under various bone density conditions could aid in selecting appropriate implant placement protocols. This study aimed to determine whether drilling torque values measured using the novel drill could serve as a guide for implant placement protocol.

**Material and Methods:**

Experiments were conducted using solid rigid polyurethane bone blocks corresponding to Misch classifications D2–D4. After measuring the drilling torque, the implant sockets were prepared under two conditions: undersized and normal-sized drilling protocols. The implant was placed, and the insertion torque value was measured as the primary stability.

**Results:**

A correlation was observed between the drilling and insertion torque values across different bone densities and surgical protocols.

**Conclusions:**

Drilling torque measurements using the novel drill may be a useful method for selecting appropriate implant placement protocols.

** Key words:**Drilling torque value, Implant, Primary stability, Undersized technique.

## Introduction

Implant treatment is a functionally and aesthetically excellent treatment in which an implant is placed into the bone to provide prosthetic restorations. Therefore, the bone density at the implant site greatly affects the treatment prognosis [1]. Bone density is closely related to the primary implant stability [2]. When the bone density is low, there is insufficient mechanical interlocking force between the implant and bone, making it difficult to obtain good primary stability [3]. When the bone density is high, it is easy to obtain primary stability [4]; however, there are problems such as overheating during the preparation of the implant socket [5].

The implant placement protocols vary depending on the manufacturer. The most common procedure involves gradually increasing the size of the placement hole preparation drill until the preparation is completed at a slightly smaller size than the implant body, after which the implant is placed. In cases of low bone density, achieving primary stability is challenging; therefore, it is recommended to use a smaller final drill diameter than standard protocols, a method known as the undersized technique [6,7]. Thus, assessment of bone density before implant placement plays a major role in the surgical protocol. Currently, the bone density at the implant site is typically evaluated using CT values or tactile sensations during implant socket preparation [8-10]. While the CT value is objective, it is an indirect method of evaluation. Tactile sensation, although direct, is subjective, leading to considerable variability depending on the operator.

To address this issue, we proposed a new method for classifying bone mineral density based on drilling torque values (DTV) using a novel measurement drill [11]. This drill allows the implant motor to record torque values during low-speed bone drilling at the initial stage of implant socket preparation, enabling direct and objective evaluation. Our previous studies reported that this drill has a spiral blade structure and a cylindrical shape with a flat tip and can classify solid rigid polyurethane bone blocks corresponding to Misch classifications D1–D4. We also reported a correlation between DTV and CT values in bovine bone, demonstrating the usefulness of this drill in bone density assessment [11,12]. Therefore, we hypothesized that analyzing the correlation between primary stability and bone density—assessed using DTV—under different bone density conditions could aid in selecting appropriate implant placement protocols. The aim of this study was to determine whether DTV, measured with a novel drill, could serve as a reliable indicator for guiding implant placement protocols.

## Material and Methods

**Figure 1:**
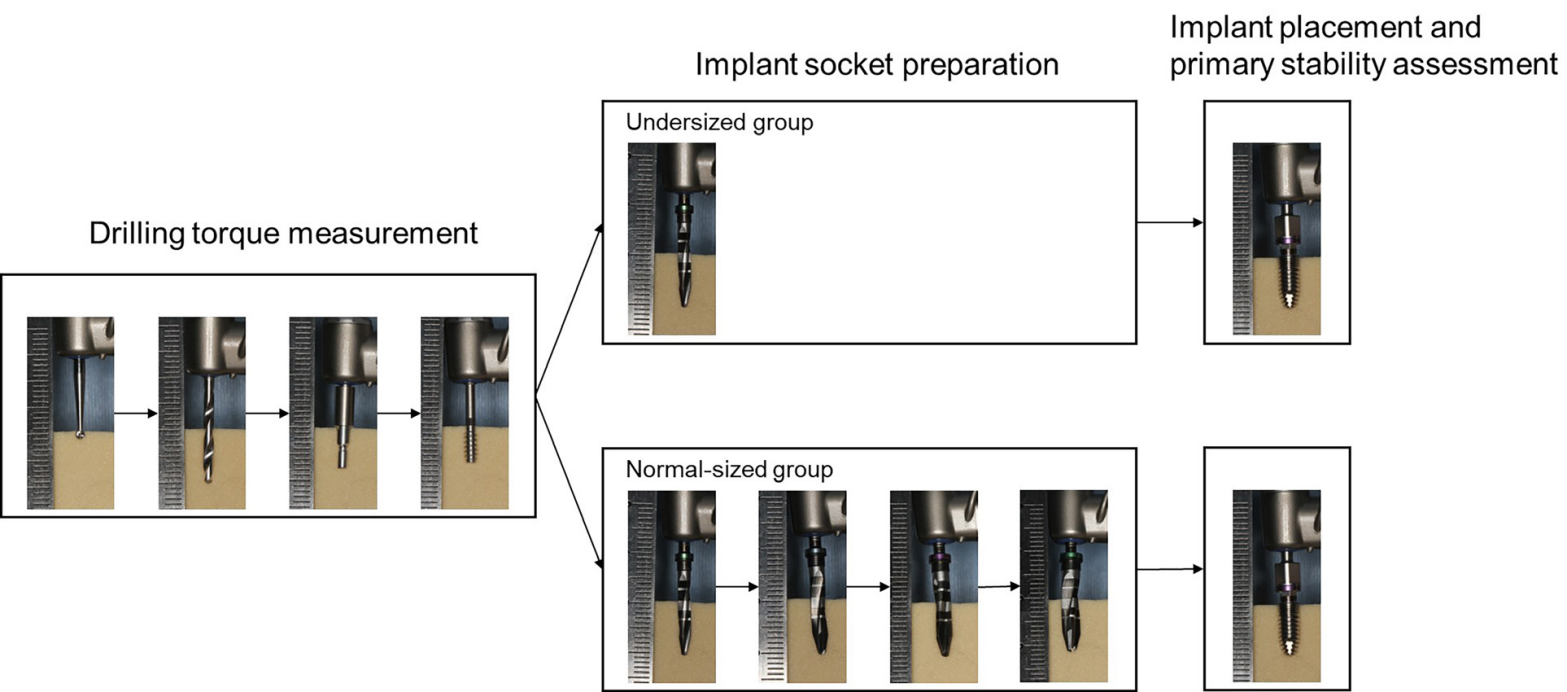
Experimental protocols for the Undersized and Normal-sized groups. The experimental procedures were divided into three phases: Drilling torque measurement, Implant socket preparation, and Implant placement and primary stability assessment. A round shaped drill, twist drill, pilot drill, and measurement drill were sequentially used in the drilling torque measurement. Implant socket preparation was performed using two drilling protocols: undersized and normal-sized drilling. Subsequently, implants were placed, and primary stability was evaluated.

In this study, two implant placement protocols were adopted: undersized and normal-sized drilling protocols (Fig. 1). The experiments were conducted using solid rigid polyurethane bone blocks (Sawbones, Pacific Research Laboratories, USA) corresponding to Misch classifications D2–D4 [13]. Ethical approval was not required for this study, as it did not involve experiments on living animals or human subjects.

After drilling torque measurement, implant socket preparation was performed by dividing the specimens into two groups: undersized and normal-sized. A micromotor system for oral surgery (Surgic Pro2, Nakanishi, Japan) was used for both drilling torque measurements and implant socket preparation. Following socket preparation, implants (diameter: 4.2 mm, length: 10 mm, machined surface, FINESIA, Kyocera, Japan) were placed, and the insertion torque value (ITV) was measured. Drilling torque measurement and implant socket preparation were performed at 1.0 cm intervals on the blocks. The measurements were performed three times by four operators (n = 12).

1. Drilling torque measurement

The DTV was measured using a method described in previous studies [11,12]. The procedure is as follows:

1) a 2.0 mm diameter round shaped drill; rotational speed, 1200 rpm

2) a 2.0 mm diameter twist drill; rotational speed, 1200 rpm; depth, 10 mm

3) a 2.8 mm diameter pilot drill; rotational speed, 1200 rpm, depth: 4 mm

4) a 2.7 mm diameter; rotational speed, 35 rpm; measurement depth, 3 mm

Since a 10 mm-long implant was used in this study, a 2.0 mm diameter twist drill was applied to a depth of 10 mm. Before using the measurement drill, saline irrigation was performed to remove the bone block debris. All procedures were conducted under continuous water irrigation.

2. Implant socket preparation

To evaluate whether intraoperative bone block density assessment using DTV allowed for the selection of an appropriate protocol, implant socket preparation was performed by dividing the specimens into two groups.

Undersized group:

Socket preparation was performed using only a final drill with 2.7 mm diameter, under continuous water irrigation at a rotational speed of 1200 rpm to a depth of 10 mm.

Normal-sized group:

Socket preparation was carried out sequentially using final drills of 2.7 mm, 3.1 mm, 3.4 mm, and 3.9 mm diameters, under continuous water irrigation at 1200 rpm, also to a depth of 10 mm.

3. Implant placement and primary stability assessment

The implant was placed at a rotational speed of 35 rpm, and the ITV was measured. The insertion torque limiter was set to 50 Ncm.

- Statistical analysis

For comparison of DTVs among blocks corresponding to D2–D4, one-way analysis of variance (ANOVA) and Tukey’s multiple comparison test were performed. An unpaired t-test was used to compare the ITVs between the two groups within each bone type. The significance level was set at *p* < 0.05. The Pearson’s correlation coefficient (r value) between the DTV and ITV was calculated.

## Results

DTVs were 2.80 ± 0.37 Ncm for the block corresponding to D2, 0.61 ± 0.17 Ncm for D3, and 0.13 ± 0.10 Ncm for D4. A significant difference was observed among all the block types.

In each block, a significant difference in ITV was observed between the two groups, with higher values associated with smaller socket diameters: D2: 27.78 ± 0.60 Ncm vs. 12.25 ± 0.92 Ncm, D3: 6.71 ± 0.32 Ncm vs. 2.73 ± 0.53 Ncm, D4: 2.84 ± 0.15 Ncm vs. 0.67 ± 0.26 Ncm (undersized vs. normal-sized, respectively) ( Table 1).

**Figure 2:**
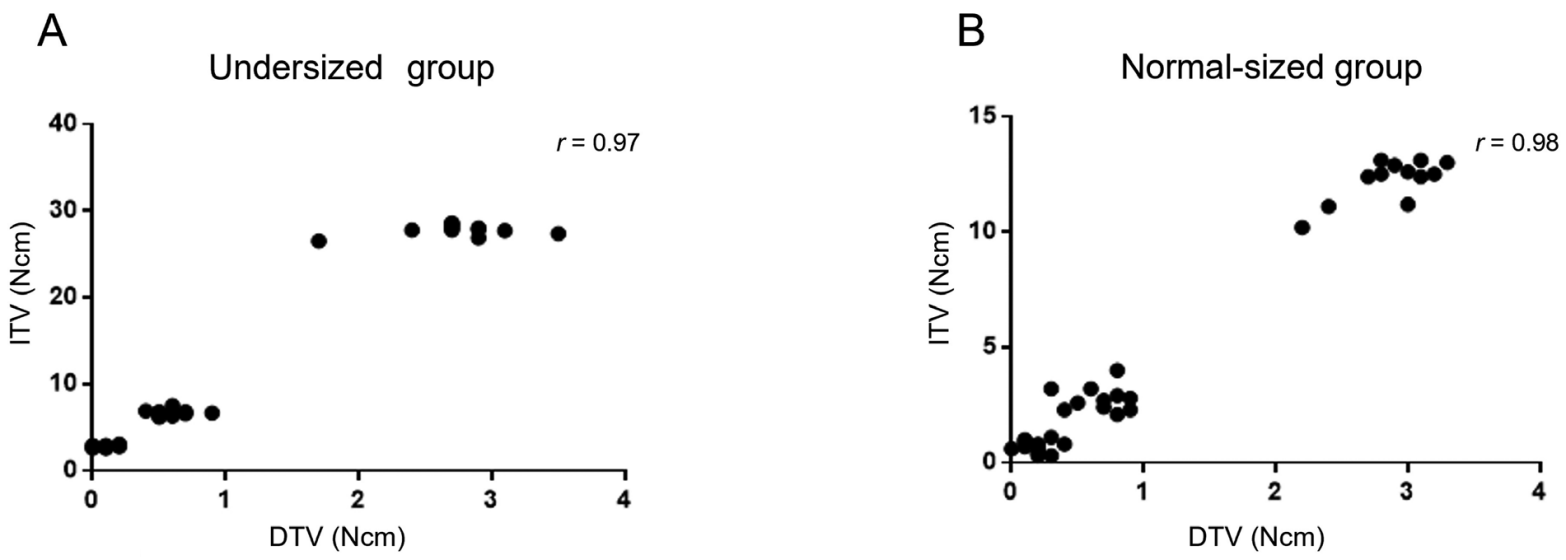
Correlation between DTVs and ITVs in each group. Correlation between DTVs and ITVs in each group. A: A positive correlation was observed between the DTVs and ITVs in the undersized group (r = 0.97). B: A positive correlation was observed between the DTVs and ITVs in the normal-sized group (r = 0.98). DTV: Drilling torque value, ITV: Insertion torque value.

Additionally, a positive correlation was observed between the DTVs and ITVs in both groups (undersized group: r = 0.97; normal-sized group: r = 0.98) (Fig. 2).

Undersized groups showed significantly higher values compared to the normal-sized groups (a > b, c > d, e > f).

## Discussion

The results of this study confirm a correlation between the DTVs measured using the novel measurement drill and the ITVs across different bone density conditions and surgical protocols.

The density measurement drill used in this study was specifically designed to assess DTVs in cancellous bones. Density assessment by DTV was performed using a 2.7 mm diameter measuring drill, following the removal of approximately 4 mm of cortical bone equivalent with a pilot drill. After the DTV was recorded, larger diameter drill was used to prepare the implant socket; therefore, bone density measurement using this drill had no effect on the subsequent implant socket preparation protocol.

The findings demonstrated that DTVs could effectively differentiate between D2-4 density blocks, which is consistent with the results of previous studies [11,12]. In this study, ITV measurements were used to evaluate primary stability. The ITV measures the resistance encountered during implant insertion. Other indicators for evaluating primary stability include ISQ; however, this was not used in experiments with cancellous bone blocks because there was no cortical bone, and the variation in ISQ values was small, making evaluation difficult [14].

ITV correlated with the normal-sized and undersized groups under conditions of D2-4. The undersized technique is a protocol in which the implant socket is intentionally prepared to be smaller than in the standard protocol. This increases friction between the implant and the surrounding bone during insertion and places the implant with high contact. As a result, the primary stability of the implant could be improved [15,16]. However, using an undersized drilling protocol in dense bone can lead to thermal injury owing to excessive friction or excessive bone compression, which may impair subsequent osseointegration [5]. Therefore, adequate bone density evaluation and subsequent decisions regarding surgical protocols for implant socket preparation are required. Previous studies have shown that bone density measurements can distinguish the resin blocks corresponding to D1-D4 [11]. An ITV between 25 and 35 Ncm is generally considered optimal for achieving favorable primary stability [17-19]. In this study, the ITV limit was set at 50 Ncm, which causes adverse effects such as bone necrosis. In preliminary verification, when implants were inserted using an undersized drilling technique in the D1 block, the ITV exceeded 50 Ncm. Degidi *et al*. reported that using an undersized drilling technique in high-density blocks resulted in an excessively high ITV [20]. Thus, in this study, we did not verify the high-density block D1. In the D2 bone blocks, the ITV of the undersized drilling protocol was 27.7 Ncm, which was within the appropriate range. For the other conditions, the ITV was < 25 Ncm. In both surgical protocols, a correlation was observed between DTV and ITV.

The ITVs were higher in the simulated bone blocks with higher densities, which also showed higher DTVs. Additionally, the difference between the preparation socket and implant diameter was a significant factor, with larger differences leading to higher ITVs.

In this study, experiments were conducted using cancellous bone blocks that did not account for cortical bones. However, in clinical settings, cortical bone is present, and therefore the ITV and DTV values may differ from those obtained in this study. Nevertheless, in cases where the cortical bone is thin and primary support must be obtained from the cancellous bone region, this measurement drill may prove to be useful.

Within the limitations of this study, a correlation was observed between the preoperative evaluation of the DTV and ITV stability. The DTVs obtained using our newly developed measurement drill were capable of directly and objectively evaluating bone density. The present study demonstrated that the DTV correlated with the ITV, and furthermore, using the undersized drilling technique improved the ITVs. Future studies are planned to include animal experiments or clinical research using *in vivo* or clinical validation methods.

The primary stability is largely influenced by the bone surrounding the implant. There is a correlation between the DTV and ITV; therefore, this evaluation method can serve as a guide for selecting an appropriate implant placement protocol that considers primary stability. In implant socket preparation, bone density evaluation by measuring DTV may be useful for selecting an appropriate implant preparation protocol.

## Figures and Tables

**Table 1:** Table Insertion torque value (ITV) (Ncm).

Misch classification	Undersized group (Ncm)	Normal-sized group (Ncm)
D2	27.78 ± 0.60a	12.25 ± 0.92b
D3	6.71 ± 0.32c	2.73 ± 0.53d
D4	2.84 ± 0.15e	0.67 ± 0.26f

Mean values ± standard deviation (SD)

## Data Availability

The datasets used and/or analyzed during the current study are available from the corresponding author.
